# The Potential of Metabolomics in Biomedical Applications

**DOI:** 10.3390/metabo12020194

**Published:** 2022-02-19

**Authors:** Vanessa Gonzalez-Covarrubias, Eduardo Martínez-Martínez, Laura del Bosque-Plata

**Affiliations:** 1 Pharmacogenomics Laboratory, Instituto Nacional de Medicina Genómica (INMEGEN), Mexico City 14610, Mexico; vgonzalez@inmegen.gob.mx; 2 Laboratory of Cell Communication and Extracellular Vesicles, Instituto Nacional de Medicina Genómica (INMEGEN), Mexico City 14610, Mexico; emartinez@inmegen.gob.mx; 3 Laboratory of Nutrigenetics and Nutrigenomics, Instituto Nacional de Medicina Genómica (INMEGEN), Mexico City 14610, Mexico

**Keywords:** metabolomics, obesity, diabetes, neurodegenerative diseases, pharmacometabolomics, exposome, extracellular vesicles, longevity

## Abstract

The metabolome offers a dynamic, comprehensive, and precise picture of the phenotype. Current high-throughput technologies have allowed the discovery of relevant metabolites that characterize a wide variety of human phenotypes with respect to health, disease, drug monitoring, and even aging. Metabolomics, parallel to genomics, has led to the discovery of biomarkers and has aided in the understanding of a diversity of molecular mechanisms, highlighting its application in precision medicine. This review focuses on the metabolomics that can be applied to improve human health, as well as its trends and impacts in metabolic and neurodegenerative diseases, cancer, longevity, the exposome, liquid biopsy development, and pharmacometabolomics. The identification of distinct metabolomic profiles will help in the discovery and improvement of clinical strategies to treat human disease. In the years to come, metabolomics will become a tool routinely applied to diagnose and monitor health and disease, aging, or drug development. Biomedical applications of metabolomics can already be foreseen to monitor the progression of metabolic diseases, such as obesity and diabetes, using branched-chain amino acids, acylcarnitines, certain phospholipids, and genomics; these can assess disease severity and predict a potential treatment. Future endeavors should focus on determining the applicability and clinical utility of metabolomic-derived markers and their appropriate implementation in large-scale clinical settings.

## 1. Introduction

**Figure 1:**
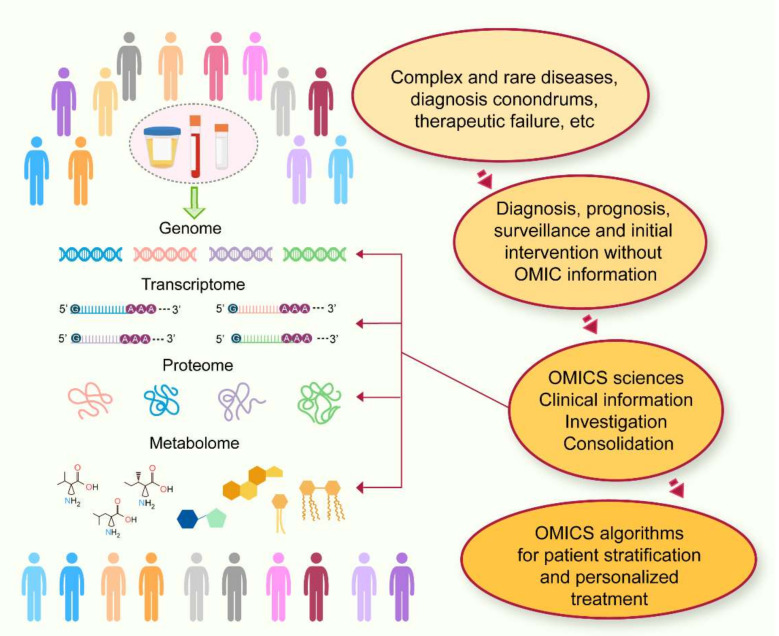
Application of metabolomics for the characterization of complex diseases. The technological advancement during the past 25 years has generated the capability to characterize the complete genome, transcriptome, proteome, and metabolome. In the clinical setting, its ability to monitor degrees of progression of complex and rare diseases is promising.

Technological developments have significantly accelerated medical discoveries. In biomedical research, many have been supported by the omics revolution. Relevant metabolic information can be tracked through the different omics levels, including genomics, transcriptomics, proteomics, and metabolomics, in an order adopted to classify biochemical processes as depicted by the central dogma of molecular biology [1]. These omic strategies, in turn, describe the layout of the biochemical organization of the human body. Genomics and transcriptomics have been extensively reviewed and focus mainly on nucleic acids [2,3]. For several decades, proteomics research has paralleled that of genomics. The advent of the Human Genome Project has boosted genomic technologies, but up-to-date comprehensive investigation of the proteome has remained challenging, both financially and technically. Metabolites and proteins can define an individual’s metabolic state by reflecting what has been encoded by the genome and modified by environmental factors at a specific point in time [4]. In this review, we focus on the metabolome, which is the closest representation and a dynamic and sensitive measure of a phenotype at the molecular level (Figure 1). The metabolome depicts the last stage of metabolism at a specific time. It has been widely accepted to be at the forefront of biomedical discoveries, whose promise is to define easy-to-measure biomarkers and to explain mechanisms of pathophysiological relevance [5].

The highly complex metabolome entails thousands of different connected metabolic reactions and, hence, small and large molecules [6]. Metabolomics has evolved as a technological tool for measuring and analyzing a wide variety of compounds, such as amino acids, carbohydrates, and lipids, from biological matrices, fluids, tissues, and a diversity of cellular fractions [7,8]. Scientists in Canada characterized and catalogued the first version of the metabolome, including 2500 metabolites, 1200 drugs, and 3500 food components [9,10]. This represented one of the first efforts to characterize the human metabolome on a large scale.

## 2. The Potential of Metabolomics in Biomedical Applications

The dissection of the metabolome provides, and will continue to provide, a collection of distinct metabolites and the integration of their profiles for medical use. This will inform clinicians, at certain points in time, about a disease’s onset, progression, or improvement. This practice will enhance diagnosis, prognosis, surveillance, and personalized drug treatments. In addition, metabolomic research has the potential to aid in the discovery of biomarkers for common and rare diseases [11]. In the next sections, we describe several applications of metabolomics in complex diseases, such as obesity, diabetes, and cancer. For rare diseases, current clinical applications already involve newborn screening that can diagnose more than 50 inherited metabolic disorders, including aminoacidopathies, organic acidemias, disorders of fatty acid oxidation, and lysosomal storage disorders [12]. A promising approach in the field of inborn errors of metabolism is the identification of disease-causing variants and its application in combination with an integrated analysis of untargeted metabolomics and whole-exome sequencing, or whole-genome sequencing. Furthermore, this strategy will expand gene–metabolite annotations [13,14]. The cell metabolome is represented by a signature of metabolites that reflect the cellular physiology at one point in time and that may or may not be representative of a disease state [15,16]. The list of interacting metabolites could hint toward molecules acting as modulators of biological processes and phenotypes and could prompt the development of new drug targets or clinical and dietary interventions [17].

Ortmayer et al. constructed a global network model across three layers of biological information: the transcriptome, the proteome, and the metabolome [18]. They explored the naturally occurring phenotypic diversity using an in vitro cell line system by integrating intracellular metabolic profiles of 54 cancer cell lines from different tissue types or in different conditions. Through this approach, they investigated the bi-directional exchange of signaling information between transcription regulators and metabolic pathways. The term transcription regulator is used for any regulator capable of modulating gene expression, including transcription factors, chromatin remodelers, and co-regulators [19]. Endogenous metabolites prone to modulate transcription regulator activity could turn into invaluable chemical scaffolds to model new therapeutic molecules targeting oncogenic regulators. The researchers found new regulatory associations of several transcription regulators with key metabolic pathways that suggest a large space of transcriptional solutions by which cells can achieve anabolic and catabolic requirements for fast proliferation and adaptation to nutrient constraints. The metabolites that affect transcription regulator activity are significantly enriched for key signaling molecules that allosterically regulate multiple enzymatic reactions, such as glutathione, glutamate, or ATP. They also observed a global coordination between glucose and one-carbon metabolism, which indicated a selective sensitivity to antifolate drugs in cell lines with low glucose uptake. This metabolic response might potentially be used as a diagnostic marker for cancer cells that are more likely to respond to folate synthesis inhibitors [18].

Metabolites have a wide range of biochemical functions, hence the growing motivation to better depict the whole human metabolome and to accelerate the generation of a comprehensive collection of clinically valid and useful metabolite profiles. This will aid investigations into in-depth, specific functions and physiological roles in health and disease, in the role of genes and metabolic pathways actionable to drug therapy or diet, i.e., pharmacogenomics and nutrition, in aging, and in acute or chronic diseases. It would be possible to improve research in metabolomics by complementing and standardizing several technologies. For example, there are efforts to fully depict a specific phenotype in time, through the creation of analytical and statistical tools to identify metabolites and metabolic pathways that are associated with particular diseases and their onset and progression [5].

One major challenge in the study of metabolomics is the existence of a substantial number of metabolites with significant chemical complexity, i.e., different functional groups, physical and chemical properties, a wide range of lipophilicity and pKa, carbon length, and chirality, among others. For example, the lipidome represents two-thirds of the plasma metabolome and consists of several thousands of lipids of at least 15 different chemical classes. Moreover, it is not fully clear if a specific metabolomic signature represents the metabolism of a specific organ, tissue, dietary intake, microbiome activity, or interaction of the microbiome with the environment [20].

Finally, this review aims to depict the current role of human metabolomics and its application in human biomedical research, including its potential to treat chronic diseases, such as cancer and diabetes, as well the likelihood of it making improvements in aging, and pharmacogenomics. Additionally, we explore the effect of metabolomics research in molecular biology, including the metabolome of the exposome and extracellular vesicles and its potential for diagnostic and therapeutic applications. Since the study of the human metabolome, unlike genomics or proteomics, must rely on several analytical platforms to tackle most of the metabolites in a clinical sample, we have also included a review of some of the analytical strategies available for this study [21].

## 3. General Strategies in Metabolomics

A thorough description of the analytical techniques used to identify small and large metabolites is beyond the scope of this review. Nevertheless, we provide a general description of the major techniques and some of their intricacies. A wide variety of matrices can be investigated from all available tissues and body fluids, including plasma [22], serum [23], cerebrospinal fluid (CSF) [24], pus [25], saliva [26], feces [27], cervicovaginal secretions [28], and urine [29]. Nonetheless, many other types of biofluids have been used in the clinic. These include sputum, bronchial washings, saliva, sweat, tears, CSF, pleural or ascitic effusions, fecal water, bile, breast milk, amniotic fluid, seminal plasma, expressed prostatic secretions, and others [30,31,32,33,34,35]. The Metabolomics Society has established guidelines for reporting details about biospecimen source, collection, and processing [36].

Broadly used for metabolomics, there are well established methods based on nuclear magnetic resonance (NMR) and mass spectrometry (MS), which can be coupled with gas or liquid chromatography, capillary electrophoresis, or ultra-performance liquid chromatography (UPLC) [37,38]. Physical and chemical properties of the metabolites of interest and their matrices will define the analytical platform for their determination.

The identification of a metabolome as a long metabolite list through an accurate spectrometry-quantification is complex due to the metabolome chemical complexity, and the dynamic range of metabolites, varying concentrations, and its challenging simultaneous quantification within complex mixtures. This causes a significant bottleneck in the field decelerating the generation of valuable biomedical information. Alseekh et al. published informative guidelines for mass spectrometry for metabolomic research, this review covers sample preparation, replication randomization, quantification, recovery, and recombination, ion suppression, and peak misidentification, as a means to enable high-quality reporting of liquid and gas chromatography (LC/GC) and mass spectrometry-based metabolomics-derived data [39].

For example, sphingolipid quantification requires prior LC/GC separation from other lipids. If double-bond information is key, then chiral columns are needed, followed by MS analysis, which may be a triple quadrupole for routine applications or a time-of-flight spectrometry, for method development or research purposes since it analyzes with a higher precision and mass resolution. Hydrophilic compounds and matrices, such as the identification of purine bases in urine, plasma, or CSF, are efficiently analyzed by NMR, but, if the interest is to identify lipids in these hydrophilic matrices, then, a chromatographical separation is needed followed by MS analysis and identification. Another key step in metabolomics is to choose between targeted or untargeted analysis [40]. The former seeks a predefined set of metabolites and can be used to identify and validate specific metabolites. The latter will detect hundreds or thousands of metabolites without a defined target list. Everything that the analytical platform can detect could be considered for further analysis. This strategy allows not only the characterization of changes in the general metabolic profile but also the detection of previously unknown metabolites thereby, promoting biomedical discovery. Here, we aim to highlight the current trends in metabolomics and the needs that ought to be addressed to implement its tools in the clinic. We have left the chemistry and analytical details out of the scope of this revision as it can be found in several seminal reports, such as those by Sanchez-Lopez, Losacco, and Theodoridis [41,42,43].

## 4. Metabolomics in Current Disease Research

The precise, consistent, and accurate assessment of myriad metabolites that may lead to biomarker discovery is still a challenge, since most matrices encompass dozens of chemically different compounds, followed by an intricate chemical or mass validation, annotation, and identification of new compounds. This list of hundreds of compounds ought to be stratified and classified for meaning and interpretation according to hypothesis-based or hypothesis-free research, in addition to the analytical strategy selected for targeted or untargeted metabolomics [44]. In this review, we describe some examples in which metabolomics has been used to search for markers of prevalent human diseases.

### 4.1. Obesity

According to the World Health Organization, obesity is a current pandemic, with 40% of adults and 340 million children being overweight or obese worldwide [45]. The major implication of obesity is that it is associated with the development of metabolic syndrome, type 2 diabetes mellitus (T2D), cardiovascular diseases (CVD), and cancer. A current challenge is to determine the metabolic changes related to the different outcomes of obesity in both humans and animal models [46]. Metabolomics has created the opportunity to establish metabolite patterns that differentiate between metabolically unhealthy obese individuals and metabolically healthy obese individuals. For example, markers of insulin resistance, a decrease in the uptake of branched-chain amino acids (BCAAs), and their accumulation in blood have been observed in obese subjects. In the case of BCAAs, their plasma concentration may be considered an early marker, since it is a feature of an increased risk of metabolic syndrome [47,48,49] (see Table 1).

Pathologic changes in adipose tissue promote peripheral inflammation, which alters the integrity of the blood-brain barrier and alters synaptic plasticity and cognitive functions [76,77,78]. These changes resemble the molecular events underlying the development of dementia and Alzheimer’s disease (AD) [79]. The increased inflammation promoted by an obesogenic environment is associated with increased catabolism of BCAAs, which has been related to insulin resistance and abnormal brain function [47]. Interestingly, decreased levels of BCAAs in the blood were associated with an increased risk of developing dementia and AD in a prospective study including eight cohorts (22,623 participants) [80]. Further research is needed to understand the interplay between obesity and BCAA levels and the mechanisms underlying cognitive impairment [81]. BCAAs, acylcarnitines (AC), and certain phospholipids have also been associated with other metabolic diseases; thus, it is important for future research in obesity to include these metabolites, characterize their biochemical pathways, and follow up with their catabolites and cometabolites [46,58].

Interestingly, the gut microbiome has also been explored in obesity [82,83,84,85]. Obesity displays a specific microbiome profile which, in turn, correlates with aspects of brain activity, including short-term memory, working memory, and changes in the volumes of the hippocampus and frontal regions of the brain [86]. The transplantation of microbiota from obese patients can decrease memory scores in mice, a feature also observed in humans. The RNA sequencing of the medial prefrontal cortex in a rodent model demonstrated a correlation between short-term memory and the expression of inflammatory genes, aromatic amino acid routes, and clusters of bacterial species [87]. These observations highlight the relevance of the microbiome and the potential relationship between therapeutically targeting the gut microbiota for memory deterioration in obese individuals [86].

An understanding of the metabolic signatures of obesity and its dynamics should lead to the categorization of obese patients to procure better characterization, improved treatment, and proper monitoring, as has been suggested by the subclassification of breast cancer and diabetes mellitus [46]. In summary, these metabolomic observations have shed some light on the physiology of the obese. Future research should focus on analyzing specific metabolites and critical pathways to address clinical challenges related to obesity, or even prevent its development and complications. This is the case of the baseline levels of BCAAs and related metabolites. They both predict and respond to improvements in insulin resistance in behavioral weight loss [31,88].

The use of polygenic risk scores, metabolomic biomarkers, and related outcomes in obesity have highlighted biological pathways, such as the BCAA pathway, that is dysregulated in this disease. These biomarkers may help in personalizing obesity interventions and mitigating of future cardiometabolic risk. A holistic approach is necessary to affect the growing epidemic of obesity and its comorbidities. For this, it is necessary to incorporate precision medicine approaches with omics strategies, such as genomic and metabolomic biomarkers, to personalize interventions and improve risk indicators.

In addition, it is important to make a metabolomic characterization of the different types of obesity and to study their relationship with comorbidities. This would allow for a better view of the most common diseases that affect humankind.

### 4.2. Diabetes

Approximately 425 million people worldwide have either type 1 (T1D) or type 2 diabetes (T2D) [45]. Thus, it is essential to apply new methodological approaches to identify molecular pathways that lead to the complications associated with diabetes. High-throughput metabolomics has contributed to the understanding of the pathophysiological pathways of T2D, including the clinical management of its complications. Several reports have shown an association between certain metabolites and T1D and T2D [89,90,91]. Most of the research in this area has used NMR or MS coupled with gas- or liquid-phase chromatography. These studies have started to reveal the metabolite changes that can help in the early identification of prediabetes, T1D, and T2D. Knowing which metabolites can modulate the effect of dietary intake and how diet affects metabolites in the body is crucial for the design of strategies, interventions, and treatments for T2D [91,92]. For example, increased levels of BCAAs and the aromatic amino acids phenylalanine and tyrosine can predict insulin resistance and T2D development [50,59,93]. Yengo et al. used a non-targeted metabolomics approach to develop a model that predicted T2D incidence 4.5% better than the known clinical markers, describing up to 90% of the parameters of the total receiver operating characteristic curve (AROC) [52] (see Table 1). Suhre et al. investigated the metabolic profiles associated with T2D and reported different metabolites depending on the tissue analyzed. For instance, they identified BCAAs in the liver and muscle tissue, cholesterol in the heart, and 3-hydroxybutirate in the blood [94]. In addition, Gudmundsdottir et al. recently reported 15 proteins as playing causal roles in T2D, including TNF superfamily Member 12, WAP/kazal/immunoglobulin/Kunitz and NTR domain-containing protein 2, growth differentiation factor 8/11, fatty acid-binding protein 4, colectan sub-family member 11, and kininogen, with several showing sexual dimorphism. This study is of relevance because it succeeded in determining which proteins had causal effects on T2D and which were affected by T2D [95].

Diabetic kidney disease (DKD), the most severe complication of T2D, and end-stage renal disease are the major causes of diminished lifespan in individuals with T2D [96]. Ibarra-Gonzalez et al., using targeted metabolomics, found that body mass index (BMI), uric acid, and tetradecenoyl carnitine (C10:2) levels were all associated with decreased estimated glomerular filtration rate (eGFR). They also found an association between albuminuria and T2D duration, A1C, uric acid, creatinine, protein intake and serum-free carnitine (C0), and tetradecenoylcarnitine (C10:2), and urinary 3-hydroxy-tetradecanoylcarnitine (C12:1) levels. DKD was correlated with age, A1C, uric acid, BMI, and carnitines C0, C10:2, and octenoylcarnitine in the plasma and C12:1 in the urine. This shows that clinical–metabolomic models increase the predictive and informative capacity to identify kidney dysfunction and DKD-related outcomes more than clinical characteristics alone [54] (see Table 1).

Similarly, Barrios et al. used serum-targeted NMR spectroscopy and found distinctive profiles in amino acid, energy metabolism, modifications of lipoprotein composition, and a broad spectrum of metabolic changes in patients with kidney disease and renal function in diabetic versus non-diabetic patients from four independent cohorts. These findings suggest that specific markers for each condition might be able to differentiate between kidney disease and renal dysfunction [55] (see Table 1).

Plasma metabolites help to predict the development of T2D [50]. For instance, in a cross-sectional study comprised of 2380 participants, it was found that a high intake of meat and low intake of whole-grain bread and tea was linked to a metabolomic signature associated with a higher risk of T2D [97]. The metabolites altered by this diet included increased hexose and diacyl-phosphatidylcholines and reduced acyl-alkyl- and lyso-phosphatidylcholines, and sphingomyelins [97].

Evidence suggests that branched-chain amino acids, ACs, and aromatic amino acids may play an early role in insulin resistance, exposing defects in amino acid metabolism, β-oxidation, and the tricarboxylic acid cycle. Diabetes risk prediction has been improved when adding metabolomic markers of dysglycemia to standard clinical and biochemical factors [98]. Metabolomics has revealed several dysregulated metabolites involved in metabolic pathways between diabetes and control samples. This knowledge could lead to multiple clinically useful biomarkers. 

We agree with Regan and Shah [99], that actionable applications in the field of metabolomics will allow for a more personalized control of diabetes, in agreement with the patient’s metabolomic profile. The clinician would be able to make decisions according to the patient’s metabolomic information, individual behavior, and demographic features.

### 4.3. Cardiovascular Disease

CVD is a general term for conditions affecting the heart or blood vessels. It is usually related to fatty acid deposits inside the arteries, hypertension, increased risk of blood clots, and vascular damage to arteries and organs, such as the brain, heart, kidneys, and eyes [45]. Predicting the risk of CVD is an important element of a preventive medical strategy. Metabolomics has helped to define changes in both global and cardiac-specific metabolism in diverse cardiovascular disease states. For example, in heart failure and ischemic heart disease, metabolomics, together with other omics, including genomics, transcriptomics, and epigenetics, have offered some insight into the molecular underpinnings of the short-chain dicarboxylacylcarnitine species (SCDA) metabolite cluster. Genomic analyses have associated SCDA levels with variants in genes that regulate components of endoplasmic reticulum (ER) stress. Importantly, these genetic variants can independently predict cardiovascular events [100]. Moreover, SCDA levels have also been associated with differentially methylated genes related to ER stress. Expression quantitative trait loci (eQTL) analyses have also linked SCDA to ER-stress pathways, specifically those reporting on the ubiquitin proteasome pathway, highlighting the relevance of integrative strategies for multiple molecular datasets [101].

The integration of metabolomics and proteomics has helped us to understand metabolic regulation of cellular processes in relation to CVD [102]. For instance, protein kinase C-delta (PKCδ), a marker of inflammation, can regulate cardiac glucose metabolism during ischemic preconditioning. These observations were made possible by the comparison of the proteome and the metabolome of PKCδ^+/+^ and PKCδ^−/−^ mouse hearts [103,104,105]. Similar studies integrating such omics have helped to increase the metabolic knowledge in atherosclerosis using apoE^−/−^ mice [106].

These integrated omic analyses have the potential to unveil mechanisms underlying the molecular remodeling in disease states. For example, a hyperpolarized 13C NMR-based approach has been used to simultaneously assess the metabolic flux across organs in a diabetic rat. Diabetes reduced the pyruvate dehydrogenase flux by 80% in the heart and 40% in the liver. The incorporation of 13C to alanine was reduced by 55% in the liver but not changed in the heart [107]. Bernini et al. (2011) used NMR and observed changes in the metabolome that correlated with CVD risk factors, such as triglycerides, low density lipoproteins (LDL), and high density lipoprotein [108]. They also identified new metabolite markers, such as 3-hydroxybutyrate, α-ketoglutarate, threonine, and dimethylglycine, but the metabolic pathways for high cardiovascular risk were shifted toward LDL, threonine, and acetoacetate.

The adrenergic signaling molecule phenylacetylglutamine in the plasma may represent a metabolite of a healthy gut microbiota. This metabolite was discovered using untargeted metabolomics and coupled with gain- and loss-of-function studies employing genetic and pharmacological data [109]. The authors revealed that phenylacetylglutamine mediates cellular events through G-protein-coupled receptors, including α2A, α2B, and β2-adrenergic receptors, which may protect against cardiovascular events [109].

The CardioNet study has reconstructed a metabolic network of human cardiomyocytes using a systems biology approach to analyze the flux balance to determine the capacity of the network to respond to different conditions of fuel supply. This network has been used to model the flux rates of substrate metabolism in working hearts under several conditions [110]. The use of these strategies will widen the capability to move from metabolomics static discoveries to dynamic models that can evaluate metabolic-based hypotheses of disorders. McGranaghan et al., in their systematic review and meta-analysis of 22 studies, found that metabolomic biomarkers, mainly lipid species, have the potential to add value to the prognosis of CVD events. Thirty-nine of the 41 metabolites were significant with a combined effect size of 1.14 (1.07–1.20) [111]. Several of these results are not conclusive; thus, it is important to replicate and validate them and to obtain a clear picture of the metabolome of CVD to generate the markers needed for its accurate diagnosis, prognosis, and treatment.

### 4.4. Cancer

Cancer has numerous effects on metabolism. These include rewiring intracellular pathways to facilitate the cancer’s proliferation and adaptation to the tumor microenvironment, and changes in normal tissue metabolism [21]. Metabolomics has the potential to identify cancer biomarkers and drivers of tumorigenesis [17]. For example, cancer metabolomics has pinpointed an upregulation of glycolysis, glutaminolysis, lipid metabolism, mitochondrial biogenesis, and the pentose phosphate pathway, as well as other biosynthetic and bioenergetic pathways [112]. In particular, in proliferating cells, mitochondrial metabolism is reprogrammed to meet the challenges of macromolecular synthesis [113]. Cancer metabolic reprogramming promotes tumorigenesis by facilitating and enabling rapid proliferation, survival, invasion, metastasis, resistance to therapies, and other central cellular processes of tumorigenesis. As tumorigenesis advances, cancer cells acquire more genetic mutations that further enhance metabolic reprogramming and, in turn, accelerate tumor growth and progression. The tumor suppressors p53 and AMP-activated protein kinase (AMPK) suppress cancer’s metabolic alterations by blocking the function, activation, and expression of essential cancer metabolic genes. For example, 14-3-3σ, a downstream target gene of p53, effectively opposes and reverses cancer’s metabolic reprogramming by accelerating the degradation of MYC proto-oncogene and bHLH transcription factor (c-Myc), a protein that promotes cancer’s metabolic reprogramming. By contrast, oncogenes, such as c-Myc, hypoxia Inducible Factor 1α (HIF-1α), rat sarcoma virus (Ras), and protein kinase B (PKB or Akt), act as major inducers of tumor bioenergetic alterations by upregulating the expression or activation of key metabolic enzymes, such as hexokinase 2 (HK2), glutaminase 1 (GLS1), and lactate dehydrogenase A (LDHA), among others [114].

Using a metabolomic strategy to classify tumors to later design customized therapies represents the most “cutting-edge” example of metabolomics enabling precision medicine [115]. Schmidt et al. analyzed a database, from The Cancer Genome Atlas, of >10,000 tumors across 32 cancer types, and they found at least one metabolic gene alteration per tumor, with a varied number of metabolic gene alterations among cancer types [21].

In-vitro models have been used to study processes, such as transformation, progression, proliferation, and metastasis. In whole organisms, metabolomic approaches suffer additional challenges and a lack of robustness for appropriate cell and tissue sampling because of the continually expanding cancer landscape. Hence, it is important to be able to discriminate between the signals of certain types of cells and those that are cancerous, in addition to being able to discern signals that are systemic. On the other hand, there are multiple challenges in the search for the identities of cell lines, as well as the standardization of culture media. In addition to looking for the Warburg effect, the citrate cycle, and lactate metabolism, it is essential to identify and develop technologies that can be used to accurately and effectively sample the heterogeneous tumor environment [44].

Another important area of opportunity for the application of metabolomics is the design of cancer treatments. For example, cancer immunotherapy has recently changed the paradigm in multiple solid and hematologic malignancies. However, the responses remain limited in a significant number of cases, with tumors developing innate or acquired resistance to checkpoint inhibition. Certain immune-sensitive tumors become immune-resistant with resultant tumor growth and disease progression. The tumor microenvironment is the environment that contributes the most to immune resistance. Nutrient deficiency, hypoxia, acidity, and the secretion of various inflammatory markers all contribute to pro- or anti-inflammatory phenotypes by modulating immune metabolism and reprogramming immune cells.

Human metabolomics in cancer immunotherapy could provide us with key molecules for understanding, predicting, treating, and controlling immune system responses. Anticipating the metabolism of immune cells would allow clinicians to implement effective dietary or therapeutic interventions and target checkpoint inhibitors [116]. It is important to realize the connection between tumor cells and immune cell compartments within the tumor microenvironment, as well as the different nutrient-sensing mechanisms and the different metabolic switches. These factors play an important role in controlling the response of the immune system against tumors, with or without checkpoint inhibitors treatment [117].

The development of tumors is extremely dependent on the neighboring tumor microenvironment, where numerous immune metabolic factors play an important role in the crosstalk, modulation, and reprogramming of infiltrating immune cells [116]. It is well known that nutrients affect the cellular activity of immune cells and the tumor microbiome. Glucose, amino acids, and lipid metabolism contribute to tumor aggressiveness and checkpoint inhibitor resistance. This highlights remarkable cellular plasticity. Amino acid metabolism is indispensable for immune-cell activation and differentiation. Tumor and immune cells compete for amino acids, such as tryptophan (trp), glutamine, and L-arginine [118]. Additionally, the role of lipids in modulating cancer-related inflammation, myeloid cells, and reprogramming has recently been recognized and will require additional research [119]. Targeting the mentioned aspects of metabolism may be key to finding the means of reversing the immune process in different kinds of cancer.

Mass spectrometry imaging has three potential applications in cancer research:

(i) the identification of next-generation prognostic and therapeutic biomarkers by establishing a chemical and morphological mapping of regions of interest,
(ii) the evaluation of the molecular efficacy of chemotherapeutic agents, and
(iii) the classification of tissue types based on molecular patterns to understand their pathways and therapeutic prognoses [120].

ACs have been associated with the early stages of different cancers. For example, the ratios of serum 3-hydroxy-octadecanoylcarnitine, (C18:2)/octadecenoylcarnitine (C18:1), and C18:3/C18:1 can differentiate early-stage pancreatic cancer from pancreatitis, with a sensitivity of 86.7% and specificity of 88.6%. An AC panel composed of decenoylcarnitine (C16:1), octadecadienyl-L-carnitine (C18:3), C18:2, C18:1, arachidonoyl carnitine (C20:4), and docosahexaenoic acid (C22:6) has shown an outstanding diagnostic ability to differentiate advanced-stage pancreatic cancer from controls and pancreatitis with an AUC value of 0.989, a sensitivity of 91.7%, and a specificity of 98.6% [121]. Furthermore, this same panel of C16:1, C18:3, C18:2, C18:1, C20:4, and C22:6 lipids and AC could differentiate early-stage breast cancer from healthy controls with an AUC value of 0.953, a sensitivity of 83.3%, and a specificity of 87.1% [56]. Similarly, a combination of C16:1, C18:2, C20:4, and C22:6, as a biomarker panel, has shown excellent diagnostic ability to differentiate early-stage colorectal cancer from healthy controls plus benign colorectal disease. Colorectal diseases are accompanied by decreased serum levels of unsaturated fats fatty acids (FFAs). This indicates that the detection of serum unsaturated FFAs might have important clinical significance for the early detection of colorectal cancer [56] (see Table 1).

The use of saliva, serum, and tumor tissues have defined metabolic signatures that enable clinicians to detect oral cancer and several tumor-specific metabolites that could differentiate oral cancer from healthy controls or precancerous lesions. These metabolic signatures, with the appropriate validation, can be used as potential biomarkers for the screening or early diagnosis of oral cancer. Future research in oral cancers should aim to build routine laboratory protocols for assessing the metabolic signatures. It should also aim to predict the metabolic responses of cancer cells to chemotherapy [122]. Hartmann et al. developed an innovative strategy for characterizing the metabolic regulome of single-cells. The strategy is called single-cell regulome profiling. It uses high-dimensional, antibody-based technologies to quantify proteins that regulate metabolic pathway activity. They applied this process to metabolically repressed cytotoxic T-cells in human colorectal carcinoma. This uncovered an abundance of metabolic regulators and found associations with metabolic flux and pathway activity. The application of this approach should enable a better understanding of human immune cell biology and help to identify disease-associated metabolic alterations that could serve as potential biomarkers and therapeutic targets for a variety of human diseases [123].

Prostate cancer is one of the most prevalent cancers and a significant cause of morbidity and mortality in men [124]. A comprehensive metabolomic analysis identified a group of metabolites that not only constitute potential biomarkers for aggressive PC but also provide molecular information about the underlying biochemical mechanisms. The information generated can be useful for the future design of diagnostic and therapeutic approaches for further validation in large patient cohorts. The detected metabolic differences between ERG-positive and ERG-negative prostate cancer demonstrate that the increment in β-oxidation and purine metabolism regularly described for prostate cancer could be principally attributed to TMPRSS2-ERG-negative (transmembrane serine protease 2 (TMPRSS2)) tumors. These results agree with the view that ERG-positive (ETS-Related Gene (ERG)) and ERG-negative prostate tumors should be considered partly different diseases, which may require different treatment strategies.

MacKinnon et al. described the metabolites involved in an androgen-dependent prostate cancer cell line [125]. Methyltrienolone (an androgen receptor agonist) treatment resulted in a metabolic signature characteristic of aggressive prostate cancer. Specifically, researchers observed a decrease in myoinositol, altered glutathione levels, a perturbation of amino-acid levels, a decreased level of methionine, a high level of phosphocholine (PC), and an increase in the phosphocholine/glycerophosphocholine ratio. These metabolites may be useful for monitoring cancer development and aggressiveness [125].

The in vivo detection of clinically relevant prostate cancer can be improved using metabolomics-derived markers related to Gleason score with non-invasive methods, as is the case for magnetic resonance imaging or positron emission tomography imaging. Analogues of PC, glutamate, and glucose, as identified here, are already applied in prostate cancer studies and have been approved by the U.S. Food and Drug Administration (FDA) for the positron emission tomography imaging of recurrent prostate cancer. The researchers discovered two additional metabolites associated with prostate cancer: hypoxanthine and arginine. Both are associated with prostate cancer recurrence and progression [57]. (see Table 1).

Although it is used less frequently than the other omics approaches, metabolomics has the potential to significantly affect core areas of oncology, including screening, diagnosis, and therapy. However, such applications require a better understanding of how these measurements are connected to human physiology and cancer biology. In biofluids that are readily accessible clinically, most notably, plasma, our understanding of which metabolites can be measured to reflect cancer status is in its early stages. Even though some incursions have been made in this field, the extent to which a metabolite profile in the plasma reveals the metabolic activity of the cancer is unclear. Additional metabolomic studies in fluids that harbor the cancer and connect these measurements to both metabolism and the biology of the tumor are necessary to understand how to interpret cancer metabolism from these measurements [21].

Metabolomic approaches may help to have a better understanding of cancer signaling [5] by providing new knowledge to develop therapeutic interventions with current drugs to target different stages and types of cancer. The actual knowledge of which metabolites can be measured to reflect cancer status is in an early stage, but some progress has been made; for instance, potential metabolomic biomarkers have been found in metabolomic urine studies in urogenital cancer [126], prostate cancer [113,114,127,128], bladder cancer [129], urinary tract infections [116,130], and pancreatic ductal adenocarcinoma [131]. In particular, lipid metabolism seems to be increasingly important in the modulation of cancer-related inflammation, expansion of myeloid cells, and reprogramming of inflammatory phenotypes [119], but follow up studies are needed. The comparison of saliva metabolomic profiles in various cancer types using capillary electrophoresis coupled with time-of-flight mass spectrometry has identified phenylalanine, valine, and leucine associated with oral, breast, and pancreatic cancer-specific profiles [132]. It is still unclear to what extent a metabolite profile reveals the metabolic activity of the cancer. Much remains to be learned about how to interpret cancer metabolism from these measurements.

Many of the mechanisms involved in tumorigenesis remain elusive; pursuing a global understanding of them using metabolomics presents many opportunities and challenges. There is a need to understand how metabolites interact and are able to influence individual cancer progression or survival. The integration of cancer metabolomics, with other omics, will allow us to better understand the several steps of cancer development, progression, treatment effectiveness, and remission. A successful example of the integration of metabolomics with other omics approaches is the study by Bonanomi. The study reported that cancer cells challenged by genetic or chemical down-regulation of the C-terminal binding proteins 1 and 2 (CtBP1,2) show significant transcriptional and metabolic plasticity regulating the crosstalk between their biochemical pathways and the cell’s demands. This could potentially cause drug resistance. This highlights the relevance of considering the effects of drug treatment when designing metabolism-based anti-cancer therapeutic regimens [133]. Further research is needed for a better understanding of how those adaptive mechanisms are connected to human physiology and pathophysiology in this field.

### 4.5. The Metabolomics of Neurodegenerative Diseases

Although the exact causes of neurodegeneration are not well defined, research efforts during the last three decades have revealed the general cellular mechanisms that underlie neuronal death. The cellular processes altered during neurodegeneration include mitochondrial function, the management of oxidative stress, the modulation of proteostasis pathways, and neurotransmission systems. It is proposed that the consequences of these alterations gradually lead to the degeneration of specific neuronal nuclei. Neurodegenerative diseases, such as Alzheimer’s disease (AD) and Parkinson’s disease (PD), are usually diagnosed when overt symptoms are displayed. This implies that neurodegenerative processes have occurred during the previous years or decades. There is a need to develop non-invasive diagnostic methods for the early detection of these pathologies. The emergence of metabolomics as a tool to analyze the dynamic interactions between the organism and environment has opened the possibility to identifying subtle chemical changes associated with AD and PD progression and the influence of the gut microbiota [134,135,136,137].

In the case of AD, there is documentation of alterations in the levels of small and lipid metabolites, in both human samples and animal models, that are related to oxidative stress, energy metabolism and mitochondrial dysfunction. In serum from AD patients, there is a decrease in oleamide levels and an increase in prostaglandin and diacylglycerols, which may be related to alterations of neurotransmitter systems and membrane integrity, respectively [66]. Evidence of mitochondrial dysfunction has been associated with increased levels of 3-hydroxyisovalerate in the plasma of AD patients [67]. Due to the complexity of neurodegenerative diseases, metabolomic information has been correlated with classical markers of neurodegeneration. For AD, extracellular amyloid-β (Aβ) deposits, phosphorylated tau, and increased levels of neurofilament light chain (NF-L) in the CSF are associated with late stages of the disease. The levels of plasma metabolite species belonging to the biogenic amine, amino acids, and AC classes are positively correlated with the levels of NF-L. This suggests disturbances in the nitric oxide pathway, neurotransmitter regulation, and mitochondrial function [68]. In addition, certain bile acid metabolites are associated with central markers of neurodegeneration, including the levels of Aβ1–42, p-tau181, and t-tau, in the CSF, as well as reduced glucose metabolism in the brain [69]. Interestingly, the levels of glycolithocholic acid and taurolithocholic acid, which are produced by bacteria, are correlated with high levels of t-tau in CSF. This indicates reduced glucose metabolism and structural atrophy [69].

Animal models offer the advantage of allowing the simultaneous monitoring of metabolite levels in different biofluids and tissues. A tauopathy transgenic rat model (SHR72) found that the brain presents increased purine nucleotide catabolism, the plasma contains decreased levels of citric-acid-cycle intermediates and glucose, and the CSF presents decreased levels of arginine and proline. Together, these data indicate impaired energy metabolism that might be connected to events of neurodegeneration [138]. Similarly, a tauopathy mouse model (rTg4510) found that changes in the levels of metabolites (glutamine, serotonin, and sphingomyelin C18:0) in the brain cortex correlate with memory impairment [70]. Future studies with animal models should validate metabolite profiles using different time points and transgenic models.

PD patients also present a different metabolite signature when compared to healthy individuals. Saliva and serum contain increased concentrations of several amino acids and other metabolites, including phenylalanine, tyrosine, histidine, glycine, acetoacetate, taurine, trimethylamine N-oxide, gamma-aminobutyric acid (GABA), N-acetylglutamate, acetoin, acetate, alanine, isoleucine, valine, cystine, proline, ornithine, fucose, propionate, and phosphoethanolamine [71,72]. These biochemical changes could result from the presence of altered or mutated proteins that have been linked to PD development (e.g., a-synuclein, leucine rich repeat kinase 2, parkin, and PTEN-induced kinase 1). The overexpression of mutant a-synuclein A53T in mice alters the brain concentration of adenine nucleotides, taurine, nicotinamide adenine dinucleotide, and metabolites from the tricarboxylic acid cycle and purine metabolic pathway [73,74]. Overall, these metabolic changes detected in PD patients and models indicate an alteration of energy metabolism and neurotransmitter regulation. The metabolomic profiling of chemical models of PD supports this notion [139,140]. The striatum of mice treated with 1-methyl-4-phenyl-1,2,3,6-tetrahydropyridine (MPTP) and lipopolysaccharide showed dysregulated levels of metabolites from the purine metabolism pathway. This highlights the possibility of adenosine deaminase becoming a promising therapeutic target [75].

Using metabolomic analysis to understand the pathogenesis and progression of neurodegenerative diseases has complemented the search of classical biomarkers that indicate advanced neuronal damage. It is expected that, in the near future, a better characterization of altered levels of several lipid species and amino acid derivatives in biofluids will reveal a list of biomarkers that would indicate risk or early phases of neurodegeneration [134,141,142,143] (see Table 1).

## 5. New Directions in Metabolomics

### 5.1. The Exposome

The exposome refers to all the environmental, biological, and physical factors that may surround an individual from conception. It includes exposures from all sources, such as radiation, stress, infections, pollution, lifestyle factors, oxidative stress, diet, social influences, geographical factors, weather, etc. [144]. Currently, an estimated 7–10% of all human diseases are attributable to environmental and occupational factors, but it is likely that this has not been fully assessed [145]. Modifying various environmental descriptors, such as diet, weight, inactivity, and smoking, has shown to reduce the risk of stroke, colon cancer, coronary heart disease, and T2D by approximately 70–90%. Therefore, the exposome may be playing a role that has not been fully accounted for [146]. Child Health and Development Studies (CHDS) provide a successful example of exposome research. They followed a birth cohort of more than 15,000 pregnant women from 1959 to 1967 in California. Researchers collected exposure data and health records in multiple generations between 2007 and 2019 and reported a link between early-life exposure to dichlorodiphenyltrichloroethane (DDT) and breast cancer risk [147,148,149,150]. Recently, Li et al. investigated the relationship between 39 environmental chemicals and the serum metabolome. These exposures only account for a few percentages of variance in the metabolome. This analysis revealed that the metabolite communities associated with the exposures were non-specific and shared among exposures; a small number of metabolic phenotypes may account for the response to a large class of environmental chemicals. This compendium of chemicals is the beginning of the exposome and a static view that excludes the possibility of disease causality to be manifested at a different time or by a different data type. Because the exposures can modify the metabolome, and vice versa, it is useful to view them as an interaction model, which can be applied to investigate outcomes, such as breast cancer [151].

The Helix Project was designed to gain an insight into the impact of multiple environmental hazards in an early life exposome. Children and mothers from Spain, Norway, Greece, Lithuania, England, and France have now completed an extensive study. There are measurements for outdoor exposures for a total of 28,000 mother-child pairs in Europe. For example, Lau et al., as part of the Helix Project, characterized the major determinants of the child metabolome in urine and serum samples from 1192 children from birth cohorts in six European countries. They correlated metabolite abundances with age, sex, BMI, and dietary habits in European children. Metabolites were measured using high-throughput 1H NMR spectroscopy and a targeted LC-MS/MS metabolomic assay. Urinary and serum creatinine were positively associated with age. Metabolic associations to BMI *z*-score included a novel association with urinary 4-deoxyerythreonic acid in addition to valine, serum carnitine, short-chain AC (C3, C5), glutamate, BCAAs, lysophosphatidylcholines (LPC a C14:0, LPC a C16:1, LPC a C18:1, and LPC a C18:2), and sphingolipids (SM C16:0, SM C16:1, SM C18:1). Dietary-metabolite associations included urinary creatine and serum phosphatidylcholines with meat intake, serum phosphatidylcholines with fish, urinary hippurate with vegetables, and urinary proline, betaine, and hippurate with fruit intake [152,153]. Population-specific variances, such as age, sex, BMI, ethnicity, diet, and country of origin, were better captured in the serum than in the urine profile. These factors explained a median of 9.0% variance among serum metabolites versus a median of 5.1% among urinary metabolites. Metabolic pathway correlations were identified, and concentrations of corresponding metabolites were significantly correlated (r > 0.18) between urine and serum (Lau CE, 2018) (http://www.projecthelix.eu; accessed on 19 November 2021).

High-resolution mass spectrometry (HRMS) has been useful in measuring the exposome; however, there is currently no universal approach to measuring it. At present, the development of analytical methods for the complete characterization of the exposome presents a challenge. It is hoped that it will provide new opportunities in the field of epidemiology to support the discoveries that will help to improve public health [154].

High-tech advances, such as HRMS and network science, have generated the first steps to enable a comprehensive evaluation of the exposome. Given the recognition of the dominant role non-genetic factors play in disease, its further evaluation has the potential to identify environmental contributors to health and disease in a manner complementary to the genome [155].

The information that will be delivered by the study of the exposome will eventually contribute to understanding the environmental causes of disease to discern between heredity and environment, or to unravel differences between nature and nurture [156]. Metabolomics has been investigating the exposome looking for a diversity of molecules related to external exposures, such as those related to diet, medication, and pollution, as well as those associated with biological responses (e.g., endogenous processes), throughout the lifespan [157,158]. The goal is to connect and associate different metabolites with metabolic modifications. This would facilitate informed choices for diagnosis and prevention [159]. The heritability of 30 of the most prevalent diseases ranged from 0.166 to 0.561 h^2^, suggesting that the exposome has a major effect on disease [32]. The vast majority of human exposures have not been explored. Research on the exposome will help to understand the effects of the environment on human health and disease. Li et al. tried to understand mixed environmental exposures as a hierarchical community between the metabolome and mixed exposures to DDT, poly and perfluoroalkyl substances (PFAS), and polychlorinated biphenyls in CHDS. Their network model revealed that most metabolite communities were not specific to the association with a particular exposure and that many are shared between exposures. That is, a small number of metabolic phenotypes may account for the response to a large class of environmental chemicals [151].

A detailed assessment of all the exposome molecules associated with health and disease may have the potential to assess and improve our environment as humans in several ways. It could provide tools to ensure chemical safety. It could challenge our current views on the origin and pathogenesis of disease. It could generate public policies and health guidelines to affect daily human health and, thus, quality of life and even life expectancy.

The investigation of the exposome is a huge endeavor. We ought to depict it by its parts: (i) the external exposure of all human factors, including the socioeconomic environment, i.e., social capital, education level, urban–rural environments, and climate factors, (ii) more specific exposures, such as stress, specific contaminants, diet, physical activity, drug consumption, allergens, and infections, and (iii) the internal exposure translated into changes in the metabolism, the immune system, and the gut microbiome. The integration of the diversity of exposures will have to consider that these are individual, dynamic, and interdependent [160].

Metabolomics is one of the most powerful tools for investigating the relation between the genetic background and the exogenous and endogenous factors within human health. In order to use this kind of complex approach, it is necessary to develop multianalyte targeted metabolomics platforms for large-scale quantitative exposome research, such as the one developed by González-Domínguez et al. They cover a broad range of chemical classes, including amino acids and derivatives, organic acids, biogenic amines, vitamins, fatty acids, acylcarnitines, and steroids (~500 endogenous metabolites). They built an endometabolome library and monitored 450 additional metabolites to obtain a representative overview of the food metabolome and other lifestyle habits. The library also includes other common xenobiotics; among them are pollutants, household chemicals, and commonly consumed drugs. This allows for a comprehensive exposure assessment. To investigate the role of microbiota in this complex interplay between external factors and endogenous processes, several microbiota-derived metabolites were considered. These included biotransformed food components, aromatic amino acid derivatives, short-chain fatty acids, bile acids, vitamins, and others. The platform simultaneously detected 1019 metabolites in short run times (<30 min. per sample). They described, for the first time, the optimization, validation, and application of a multi-metabolite platform for comprehensive and quantitative metabolomics-based exposome research. This approach is of great utility in diverse research fields, such as health research, nutrimetabolomics, toxicometabolomics, and pharmacometabolomics. This kind of approach is challenging because of its complexity. Despite that, this research has several strengths; for example, it has the ability to simultaneously quantify more than 1000 metabolites in different types of samples, in short-run times, by using simple and automatable extraction methods, and using small-sample volumes. This facilitates its implementation in large-scale epidemiological studies [161]. This kind of metabolomic approach should be implemented in diverse fields of biomedicine to address the complexity of the exposome, thus helping to improve human health.

### 5.2. Pharmacometabolomics

The interindividual variability in drug response and safety has prompted investigations to search for markers to predict and personalize drug prescription, minimize toxicity, and detect a lack of efficacy. In the last 70 years, pharmacogenetics and pharmacogenomics (PGx) have converged in the publication of 26 PGx guidelines officially approved for directing drug prescriptions for more than 60 drugs. Their clinical implementation is paving the way to a clear strategy for identifying useful gene–drug associations. However, adverse drug reactions represent the fourth leading cause of death in the USA, and PGx makes 72% of these preventable [162]. Similar statistics have been reported for the UK [163], Spain [164], and Italy [165]. The incidence of adverse drug reactions will only increase as the population ages and the number of drugs prescribed increases. This points toward a need for the identification of better tools for personalizing drug treatment. PGx has identified genotypes associated with drug responses for about 60 drugs, and the FDA has approved around 3000 drugs. Therefore, several hundred drugs still need markers to improve their efficacy and safety, especially those with narrow therapeutic indices.

A metabolomic profile reflects the impact of genetic and environmental factors on a certain phenotype. This brings us closer to a real-time phenotype capable of predicting the actual state of a patient’s drug response. Weinshilboum et al. agreed that metabolomics can inform pharmacogenomics to ultimately identify genetic variants of more specific phenotypes [4]. Beyond genetics, it is within the scope of pharmacometabolomics to pinpoint metabolites for drug monitoring and personalized medicine in addition to collecting genetic information. Metabolomics can offer a more immediate metabolic status whose application has been widely acknowledged for newborn screening programs around the world, although technology has been the limiting factor for its broader implementation. Metabolic information has already guided drug response. For example, clinicians measure the presence of receptor on the surface of some cancer cells (HER2) biochemically (not genetically) prior to prescribing trastuzumab. Some physicians prefer thiopurine methyltransferase (TPMT) phenotyping to genotyping for the administration of thiopurines as the former may be more precise for identifying potential toxicities [166]. Changes in oxidative states in health versus disease, or due to drug treatment, are rarely adequately defined by genomics, whereas metabolic assessments can provide a comprehensive picture. In a recent case study, the metabolome of a patient suffering from a rare cancer and recurrent infections showed significantly higher homocysteine/methionine and homocysteine/thiodiglycolic acid ratios compared to that of healthy aged-matched controls. These differences highlighted a lower antioxidant capacity, guiding medical decisions toward a personalized protocol involving a 10-fold increase in vitamin C supplementation, which improved the efficacy of anticancer drugs and antimicrobial control [167].

Another example showed that, during cancer chemotherapy, the accumulation of AC and specific amino acids partly reflects a patient’s exposure to SN38, the most abundant, active, and toxic metabolite of irinotecan [60]. Consequently, a threshold level of AC could be used to assess and monitor irinotecan’s dosing and toxicity risk. Similarly, the interindividual variation in the response to aspirin’s antiplatelet effects is likely due to plasma variations of amino acids and serotonin levels. Measuring these metabolites could prevent hemorrhages and increase antiplatelet efficacy in high-risk patients [168]. Metabolic signatures involving amino acids, carbohydrates, and lipids already provide information differentiating between gemcitabine-resistant and sensitive pancreatic cancer patients. The above shows that metabolomics is a key complement for determining drug safety and efficacy and that support for research and development in the field ought to be granted.

Moreover, metabolomics research on pharmacokinetics and pharmacodynamics may guide drug development, efficacy, and safety. Alterations of the metabolic profile of a disease can be due to the disease itself, the patients’ genotype, or the drug’s effects. This supports the notion that we need metabolomic strategies to determine each stage of progression from the exposome, to health and disease, to treatment efficacy. Lipidomics, for example, has been used to identify lipogenesis inhibitors that target specific lipids that may be used in anticancer treatment to hinder the progression of cancer [61]. However, care should be taken when considering metabolic profiles as biomarkers of drug efficacy for prognosis since most diseases are still limited in their molecular characterization, and new disease subtypes are being characterized every day. In this regard, Koch et al. identified a list of glutaminolysis-related metabolites whose decrease indicates drug effectiveness against glioblastoma stem-like cells, but the inhibition of glutaminolysis was observed only for glioblastoma stem-like cells, a cell subpopulation in glioblastoma [169]. In addition, as technology evolves and prices decrease, the personalization of drug prescription will comprehensively and individually improve to assess genomics, metabolomics, the exposome, the microbiome, and other omic strategies. The field of pharmacomicrobiomics can inform clinical pharmacogenomics, as the microbiome has been associated to drug response, the pharmacokinetics and pharmacodynamics of several drugs, including metformin [64], antibiotics [62], antihypertensives, and anticancer immunotherapeutics [63].

Future endeavors in pharmacometabolomics will seek to understand drug safety and efficacy by generating a metabolomic and genomic signature for each medication. This is particularly urgent for illnesses related to aging, such as diabetes, hypertension, dyslipidemia, cancer, and mental diseases. This will only be attained as technological advances facilitate access to metabolomic analyses and statistical platforms enable clinical testing integrating biomedical research.

### 5.3. Metabolomics and Extracellular Vesicles

The relevance that extracellular vesicles (EVs) have gained in the past few years lies in their potential for diagnostic and therapeutic applications. EVs comprise a heterogeneous group of membrane-bounded nanoparticles released to the extracellular space that reflects the physiological state of the cell of origin. The two main types of EVs involved in cell-to-cell communication events are generated through the invagination of the membrane of the multivesicular bodies of the endosomal pathway (i.e., small EVs and exosomes) and through the outward budding of the plasma membrane (i.e., large EVs, ectosomes, or microvesicles). Both types of EVs transport RNA, proteins, lipids, and metabolites that influence the functioning of recipient cells. Alterations in their molecular cargo have been documented, especially through transcriptomic and proteomic approaches in several pathologies, including cancer and metabolic, cardiovascular, and neurological diseases. Recently, the analysis of lipids and small metabolites has proven to be a useful strategy for identifying biomarker candidates that cannot be measured directly in biofluids [170,171,172].

Small and large EVs display a metabolic profile that is highly correlated with their parental cells [170,173]. Thus, lipidomic analyses are a useful strategy for identifying novel biomarkers and determining the source of circulating EVs. An analysis of the lipid composition of serum-derived EVs from healthy individuals revealed the existence of different EV subpopulations distinguished by their phospholipid or ceramide contents and protein markers [174]. Similarly, urinary small EVs from healthy individuals show a unique content of diverse types of sphingolipids, excluding phosphatidylinositol ceramides, which were detectable only in large EVs [175]. In response to tissue damage, there is a dysregulation of specific metabolic markers in EVs that cannot be traced in whole plasma or serum. For instance, after cranial irradiation in a mouse model, there is an increase in plasma-derived EVs of inflammatory markers, including triglycerides, platelet activating factor (PAF), carnitine, and C-16 sphinganine [176]. The transport of inflammatory mediators has also been confirmed in EVs from humans. Pro-inflammatory metabolites, such as prostaglandin F2-alpha (PGF2α), are transported in the plasma and have been related to an increased risk of thrombotic events in patients with paroxysmal nocturnal hemoglobinuria [177].

The metabolomic characterization of EVs in the cancer field has been directed toward determining if there is a molecular fingerprint associated with the degree of aggressiveness of the tumor and to predicting on drug sensitivity. These studies have confirmed a specific enrichment of lipids and metabolites that is distinguishable from cell metabolomes [178,179,180]. Moreover, it seems that there is a molecular signature that allows the differentiation of metastatic cell lines from primary tumors. The EVs from colorectal cancer cell lines show an alteration of glycerophospholipid subtype levels and a shift of arachidonic and docosahexaenoic acid to diacylphosphatidyl-ethanolamine plasmalogen, according to the degree of malignancy [178]. In prostate cancer cell lines, there is also evidence of a lipidome signature for metastatic cell lines. The PC-3 cell line, which is highly metastatic, generates vesicles enriched in sterol lipids, sphingolipids, glycerophospholipids, and monounsaturated fatty acids [181,182]. These differences in lipid content affect tumor progression. EVs derived from highly metastatic breast cancer with increased levels of unsaturated diacylglycerol species stimulate angiogenesis through the activation of endothelial cells [180]. Moreover, the drug resistance of tumors has been associated with the presence of a particular phospholipid signature of EVs from lung cancer cell lines [183].

In addition to transporting metabolites that directly affect the physiology of target cells, EVs contain a variety of enzymes that confer the ability to alter metabolite concentrations in the blood [184]. Hepatocyte-derived EVs contain the enzyme arginase I, whose levels increase under hepatotoxic conditions, both in vitro and in vivo. These hepatic EVs alter the oxidative environment in the bloodstream and regulate endothelial function through the modification of the levels of arginine and ornithine [185]. These studies highlight the need to determine the relevance of metabolic enzymes that have been documented in the proteome of EVs derived from multiple cell lines and patient samples.

Although the metabolomic analysis of EVs requires further standardization and validation, there have been significant advances in the identification of relevant biomarkers [186]. The levels of phenylalanine, leucine, phosphatidylcholine 35:0, and sphingomyelin 44:3 in large pleural EVs help to differentiate tuberculosis pleural effusion from malignant pleural effusion [172]. The abundance of ceramides, ceramide-phosphates, phosphatidylglycerol, and sphingomyelins allows for the differentiation of healthy subjects from asthmatics. It can also identify lipid metabolites that exacerbate inflammation [187]. Regarding the prediction of preterm birth, low levels of phosphatidylserine (PS 34:0), PS (O-42:0), phosphatidylinositol (O-36:1), C24 (OH) sulfatide, and phosphoethanolamine (O-33:0) in plasma-derived EVs are sufficient to distinguish preterm birth from healthy pregnancy in the early second trimester [188]. Future studies should address the functional implications of changes in the EV metabolome and compare its diagnostic and prognostic informative value relative to the information obtained from the transcriptome and proteome of EVs. The challenge is to determine whether EV metabolome would reveal unique biomarkers that indicate functional alterations in diverse pathologies and escape from analysis of unfractionated biofluids [186,189].

### 5.4. Metabolomics and Longevity

According to the United Nations, human life expectancy is still increasing. Japan and several developed countries have an average lifespan of more than 80 years. Longevity is a complex trait that is difficult to predict, and its high interindividual variability has encouraged researchers to analyze the biology underlying aging with the goal of predicting lifespan and mortality risk, as well as improving the quality of human life. Human aging is accompanied by a gradual decline in physiological functions. This has been the focus of a plethora of studies, from genetics to biochemistry, animal models, and, more recently, metabolomics [190].

The genomics of aging include the investigation of genes related to DNA repair, ROS, apoptosis, epigenetics, autophagy, mitochondria, adduct formation, and telomere protection. Genetic variation on *TOR, AKT, FOXO, APOE, SIRT1/6*, and several others has shown to impact life expectancy in animal models [191,192,193]. Currently, genomics can predict the heritability of longevity between 15 and 33%, although recent calculations barely reached 10% [194]. Consequently, other approaches have been explored to better depict and predict longevity. Calorie restriction, for example, did not demonstrate a direct impact on life extension but showed improvements on healthy aging, leaving many questions to be answered for humans [195]. More recently, metabolomic profiling has made some progress in identifying pathways for healthy aging, disease delay, and the physiological status of the long-lived. A thorough review by Parkhitko et al. included at least six independent pathways, such as glucose catabolism and anabolism, amino acids, the pentose cycle, the Krebs cycle, and pathways of mitochondrial function, that can describe metabolic activity and lifespan across species [196]. The shift in reporting metabolomic signatures instead of metabolic cycles and physiological pathways would offer a broad and comprehensive understanding of the aging phenotype by pinpointing specific compounds or a useful metabolomic signature embedded in a whole organism. 

The wide array of physicochemical properties of the metabolome have guided its analytical detection and quantification, so that progress in the field is reported separately, according to the instrumental/technological methodology. Amino-acid profiling, lipidomics, and glycomics preferentially use liquid chromatography coupled with triple quadrupole or time-of-flight mass spectrometers with a diversity of separating columns. To analyze this signature, we could suggest different platforms; free fatty acids can be derivatized and detected using gas chromatography and mass spectrometers, hydrophilic metabolites may rely on NMR or capillary electrophoresis, and, for amino acids or proteins, one could use ion trap mass spectrometers coupled with liquid chromatography. The output of these strategies is currently analyzed independently as metadata, generating long lists of organic molecules and developing testable hypotheses on human longevity or mortality risk. For example, animal models present some evidence of amino acid alterations affecting the lifespan of marmosets [197]. In C. elegans, a signature of metabolites related to phosphocholine, protein biosynthesis, the urea cycle, glutathione, amino acids, betaine, and biotin metabolism has been significantly associated with a longer life span. This may be due to an increased activity of the Krebs cycle and augmented phosphocholine levels, as well as decreased nucleotide metabolism [198]. A metabolomic profile, including vitamin E, betaine, and other polyamines, was observed in higher concentrations in the naked mole rat, an iconic animal model of longevity compared to the short-lived mouse [199]. Proteomics has delivered 25 peptides associated with longevity in men. These peptides are present in inflammation and immunity processes, including C-reactive protein, the complement components C7 and C9, immunoglobulin heavy constant mu chain (IGHM), neuropilin-1, alpha-2-macroglobulin, and cell surface glycoprotein MUC18, among others [200]. Similarly, glycomics has listed N-glycans as inflammaging markers [201], while lipidomics, the largest part of the metabolome, has identified specific ether-phosphocholines and plasmalogens with antioxidant capacity, serving as promoters of cardiovascular health and healthy aging in women. All these studies highlight the importance of a detailed profiling of individual small metabolites, amino acids, nucleic acids, and protein, and lipid species not yet broadly achieved nor integrated [51,53]. To consider the whole metabolome and its association with longevity or mortality risk, it is critical to tackle several analytical platforms simultaneously for a diversity of populations or to perform metadata analyses. For the latter, Fischer et al. developed a model to predict the short-term risk of death from all causes using only four plasma metabolites: albumin, very low density lipoprotein (VLDL) particle size, alpha-1-acid glycoprotein, and citrate [202]. This study was recently validated in 44,148 Europeans. It listed 11 metabolites, including the total lipids in chylomicrons, VLDL and small HDL, the mean diameter for VLDL particles, the ratio of polyunsaturated fatty acids to total fatty acids, histidine, leucine, valine, and albumin, for which a higher level was associated with decreased mortality. Higher levels of glucose, lactate, isoleucine, phenylalanine, acetoacetate, and alpha-1-acid glycoprotein were associated with increased mortality [65].

Noteworthy are the differences in the results and theories published depending on the study design. Some studies compared familial longevity to individual longevity, while others compared nonagenarians to centenarians, or males to females, proteomics versus lipidomics, glycomics versus NMR hydrophilic metabolomics, targeted and untargeted metabolomics, and their overlapping associations. Moreover, all the studied populations lack age-matched control groups, making it difficult to consolidate a metabolic profile [203]. To date, there are a few potential markers of healthy aging, such as lipoproteins, amino acids, and ether phospholipids, that may have biomedical utility in the near future. The coming decades will see the development and validation of tools to comprehensively integrate chemically distinct metabolite signatures from a variety of analytical platforms and different cohorts for the prediction of mortality risk, longevity, or healthy aging and their implementation for precision medicine applications [204]. Finally, these approaches will only be enriched as they develop with the consideration of additional determinants of longevity, such as diet, the microbiome, mental health, and the interactions among them [205]. This may, ultimately, serve to deliver models of mortality risk scores using succinct but specific information from diet, human and microbial genomics, and metabolomics.

## 6. Discussion

The discovery of metabolomic signatures for health and disease will deliver biomedical applications to facilitate the efficient differentiation of pathophysiological states, diagnosis, personalized treatment, and drug development. Human metabolomics research is focused on depicting, in real time, small and large molecules that characterize health and disease states. The identification of metabolomic signatures could lead to prevention and better health care treatment. Metabolomics research encompasses a wide variety of analytical tools and samples, mostly based on the physicochemical properties of the biological matrices and the metabolites of interest [206]. Metabolomics analyses can be targeted or non-targeted, hypothesis-free or hypothesis-generating, with the purpose of discovering, defining, validating, and implementing molecular signatures to aid in the progress of medicine.

Current efforts are directed at integrating metabolomic data from different sources to obtain a broad and comprehensive phenotype of a specific health or disease state. Sources include the following:

(i) different analytical platforms, such as NMR, gas chromatography, liquid chromatography, and MS;
(ii) various identified metabolites, such as lipids, amino acids, proteomics, glycomics, etc.;
(iii) different matrices, such as blood, plasma, CSF, urine, tissue, tumors, etc.

It is also important to consider that the metabolome varies with time, pathology, developmental stage, progression, drug treatment, dietary intervention, environmental factors, and even the microbiome. It is acknowledged that the simultaneous and accurate analytical quantification of the metabolome remains a challenge [21].

The integrated use of metabolomics, together with other omics technologies, will overcome some of these challenges and will determine a better pattern of recognition and association of a comprehensive human phenotype. This also presents mathematical and statistical difficulties, as a wide array of data from several omic sources ought to be integrated [207]. One example of successful omics integration is in T2D, where genomics and metabolomics, together with pathway analysis, identified BCAA levels as potentially causing diabetes mellitus. First, metabolomics identified the BCAA levels associated with the pathway of the enzyme, alpha-keto acid dehydrogenase (BCKD) complex, as the rate-limiting step in BCAA catabolism. Next, the PPM1K gene, a mitochondrial phosphatase gene, was associated with the formation of BCAAs, leading to the relationship between these three [208]. The availability of multi-omic data does not always allow for direct conclusions on disease or phenotype causality. Nevertheless, integrated omics have demonstrated several layers of evidence confirming and validating independent results directed at the consolidation of biomedical hypotheses [209,210].

Progress in analytical techniques is also warranted for metabolomics to fully emerge as a clinical tool. Classical metabolomics requires large sample volumes, from milliliters to microliters, which imposes a clear difficulty for certain biofluids [211]. Fortunately, novel strategies, such as triboelectric nanogenerator inductive (TENGi) MS, can work in the subnanoliter volume range. In fact, it has been used in a targeted and non-targeted mode and was useful in analyzing of exosomes, tissue-derived cells, needle biopsies, tears, and sweat, all of which can be collected in small amounts. It seems that TENGi MS coupling would further increase metabolite coverage and can be applied to almost every type of sample where the amount collected is limited [211].

In addition to the development of better analytical platforms, there is a need for the global standardization of metabolomic analyses. As has happened for clinical studies, drug development, genomics, and pharmacogenomics in the past, metabolomics currently lacks official protocols for reporting and validating discoveries. Hence, a unified consensus as to how to define a clinically useful and valid metabolomic signature is still missing. Schmith et al. commented that there is a need for the use of standardized protocols, low-cost affordable instruments, and user-friendly analytical platforms so that metabolomics can be broadly used in research and implemented in clinical laboratories to meet the increasing demands for diagnostic and prognostic tests [21]. Moreover, the algorithms involved in pathway analysis also require the standardization of methods, sample preparation, instrument specifications and settings, and quality controls. In this regard, OmicsDI (www.omicsdi.org) may address some of these intricacies. OmicsDI is an open-source platform that integrates and stores a diversity of omics data, publicly available for download and analysis [212]. This repository promotes data-driven research, thus diminishing redundancy in experiments. Future efforts should tackle computational and statistical challenges to homogenize metabolomic analysis and reporting, to capitalize on the promise offered by integrated metabolomics and systems biology [213].

Ultimately, integrated omics analyses aim to establish genome-scale metabolic models combining phenotype data for every disease or health state. Systems biology can already provide maps of metabolic networks in physiological systems. Linking it with actual metabolomic data will not only validate previous reports but will also consolidate current theories and hypotheses for a better understanding of tissues, organs, or even whole organisms in human disease and health [214].

The most prevalent health problems today are chronic diseases and those of an aging population. These include T2D, as well as cardiovascular and neurodegenerative diseases. Metabolomics may improve the treatment of these diseases, as well as improve longevity itself and help to personalize drug treatment. To date, there are several validated metabolomic signatures for T2D and its complications, including kidney disease and cardiovascular and neurodegenerative diseases. Metabolites, such as VLDL, AC, BCAA, mono- and polyunsaturated fatty acids, and ether phospholipids, have been consistently reported for the last decade as key metabolites of T2D, CVD, and neurodegeneration. These metabolites have been consolidated and, even validated, as specific metabolomic signatures. Next steps include finding ways to implement metabolomic signatures in clinical practice and finding a better cost-benefit balance so that metabolomics can be used to improve diagnosis, prognosis, and treatment.

The diverse, complex, and sensitive nature of the metabolome means studies should be consistent, with reduced variation between subjects. They should also prioritize information delivery and reports.

Metabolomics has provided some clear markers of disease progression and drug efficacy. For example, ACs and aromatic amino acids in insulin resistance, specific phosphocholines in cardiovascular disease, an increase of drug metabolites in risk of adverse drug reactions, and long chain sphingolipids and polyunsaturated fatty acids in female longevity. Current endeavors ought to replicate these observations and validate them to provide, in the near future, a list of useful and clinically valid metabolomic signatures to improve human health.

## 7. Conclusions

Metabolomics continues to make important contributions in biomedical research, such as the identification of markers for diagnosis, disease monitoring, and drug efficacy, all of which improve the patient’s quality of life. Analytical techniques have broadly assisted with the identification and, in some instances, are used in the clinic. The main limitation of any metabolomic analytical platform is that it cannot characterize the whole phenotype. Therefore, it seems that future metabolomic goals will include the development of more comprehensive analytical platforms and the integration of metabolomic data from different instruments. It is possible that future technological advances will create instruments that can analyze all or most metabolites, regardless of their chemical nature, requiring only one instrumentation setting. Eventually, multi-omic approaches will enable a more detailed molecular understanding of human metabolomics in health and disease. This is essential for guiding novel diagnoses and therapies [215]. Novel or integrated analytical techniques, such as metabolite imaging, statistical, and computational algorithms, are urgently required for metabolomics to emerge as a tool with analytical validity and clinical utility.

## Figures and Tables

**Table 1:** Selected metabolomic profiles in biomedicine.

Species	Metabolomic Platform	Trait	Fluid/Tissue	Refs.
Diabetes				
Phe, Gly, diacyl-phosphatidylcholines SM(C16:1), acyl-alkyl-PC, etc.	Metabolomics, LC-MS	Predictive of T2D	serum	[50]
PC (34:2), PC (36:2), TG (52:1), long chain PUFA, total TG, ceramide (22:0)	Lipidomics, LC-MS/MS	Associated with T2D	plasma	[51]
Ile, Phe, Ser, Tyr, Gly, palmitoyl SM stearoylcarnitine, etc.	Metabolomics, GC/MS LC/MS/MS, fatty acids	Predictive of T2D	plasma	[52]
Leu, Ile, Val, γ-glutamyl-derivates, PC aa (OH, COOH) C28:4, etc.	Metabolomics, NMR, GC- MS, FIA-MS, LC/MS	Associated with T2D	plasma	[53]
Diabetes kidney disease				
C8:1, C10:1	LC-MS	Increases prediction clinical models	blood/urine	[54]
C0, C10:2 and urinary C12:1 s	LC-MS	Albuminuria	urine	[54]
Gly, Phe, citrate, glycerol	NMD spectroscopy, amino acids, metabolites	Negatively associated with eGFR	urine	[55]
Ala, Val, pyruvate	NMD spectroscopy, metabolites	Positive association	serum	[55]
Cancer				
C16:1, C18:2, C20:4, and C22:6	CBDI- nanoESI-FTICR MS, FFA	Colorectal cancer diagnosis.	serum	[56]
PC, Glu, Arg, hypoxanthine, α-glucose	Metabolomics, NMR, LC/MS	Prostate cancer	tissue	[57]
Obesity				
Arg, Leu/Ile, Tyr, Val, Pro	MS/MS	Childhood obesity and serum triglycerides	serum	[58]
Leu, Ile, Val, and Tyr	Metabolomics, NMR	Abdominally obese females	serum	[59]
Val, Phe, Tyr, and Gln	Metabolomics, NMR	Insulin resistance	serum	[59]
BCAA catabolites		Insulin resistance and abnormal brain function	serum	[47]
Pharmacometabolomics				
ACs	Metabolomics, HILIC LC-MS/MS	Elevated in irinotecan exposure	plasma/serum	[60]
SM, dihydroceramide, PC, PS, PE, cys	Metabolomics/LC-MS/MS	Higher in lorlatinib treatment	plasma/serum	[31]
Palmitoleate (C16:1n-7), DHA; 22:6n-3 and EPA; 20:5n-3	Lipidomics/LC-MS/MS	Associated with fish oil antiobesity effects	plasma/serum	[61]
*Proteobacteria and Firmicutes*	Microbiome/metagenomics	Associated to beta lactam antibiotic resistance	feces	[62]
*Akkermansia muciniphila*	Microbiome/metagenomics	Increased efficacy of programmed cell death 1 protein (PD-1) immunotherapy	plasma/feces	[63]
*Escherichia coli*	Microbiome/metagenomics	Associated to metformin efficacy and toxicity	plasma/feces	[62]
*B. thetaiotaomicron*	LC-MS/MS, microbiome analysis	Diltiazem and 46 different drugs	plasma/feces	[64]
Longevity				
PC (O-34:3, O-34:1, O-36:3), SM (d18:1/14:0), PE (38:6)	Lipidomics, LC-MS/MS,	Familial longevity, higher in females	plasma	[51]
Lipids in chylomicrons, VLDL HDL, VLDL size, PUFA, Val, histidine, Leu, and albumin	Metabolomics LC-MS/MS	Longevity, decrease mortality	plasma	[65]
Alzheimer’s disease				
Prostaglandin, diacylglycerols and oleamide	Lipidomics, LC-MS/MS	Altered NT systems & membrane integrity	serum	[66]
3-hydroxyisovalerate	Metabolomics, NMR	Increased plasma levels; mitochondrial dysfunction	plasma	[67]
Biogenic amine, citrulline, Pro Arg, Ala, Thr, ACs	Metabolomics, LC-MS/MS	Nitric oxide pathway alterations; mitochondrial function	plasma	[68]
Bile acid metabolites, glycolithocholic acid taurolithocholic acid	Metabolomics, LC-MS/MS	Reduced glucose metabolism in the brain & structural atrophy; levels associated with Aβ1–42, p-tau181, t-tau	bile, serum	[69]
Gln, serotonin, and sphingomyelin C18:0	Metabolomics, LC-MS/MS	Memory impairment	brain cortex	[70]
Parkinson’s disease				
Phe, Tyr, His, Gly, acetoacetate, taurine, TMAO, GABA, N-acetylglutamate, acetoin, acetate, Ala, Ile, Val, Cys, Pro, ornithine, fucose, propionate, and PE	Metabolomics; UPLC-MS, NMR	Disease onset	serum, saliva	[71,72]
Tricarboxylic acid cycle and purine pathway metabolites	Metabolomics, LC-Ms, GC-MS, UPLC-MS	Alteration of energy metabolism and neurotransmitter regulation	whole brain, striatum	[73,74,75]

FFA: free fatty acids, PUFA: polyunsaturated fatty acids, TG: triglycerides, PC: phosphocholine lipid species, PS: phosphoserines SM: sphingomyelins, PE: phosphoethanolamine species, DHA: docosahexaenoic fatty acid, eicosapentaenoic fatty acid: EPA.

## Abbreviations

1-methyl-4-phenyl-1,2,3,6-tetrahydropyridine (MPTP), 3-hydroxy octadecanoylcarnitine (C18:2), 3-hydroxy-tetradecanoylcarnitine (C12:1), 6-hidroxidopamine (6-OHDA), acido docosahexaenoico (C22: 6), acylcarnitines (AC), adverse drug reactions (ADRs), alanine (ALA), Alzheimer’s disease (AD), AMP-activated protein kinase (AMPK), amyloid-β (Aβ), androgen-dependent prostate cancer cell line (LnCAP), apolipoprotein E (APOE), arginine (ARG), arachidonoyl carnitine (C20:4), area under the curve (AUC), branched-chain amino acids (BCAAs), benign colorectal disease (BCD), blood–brain barrier (BBB), body mass index (BMI), capillary electrophoresis (CE), cardiovascular diseases (CVD), cerebrospinal fluid (CSF), checkpoint inhibitors (CPI), Child Health and Development Studies (CHDS), cysteine (Cys), colectan sub-family member 11 (COLEC11), C-terminal binding proteins 1 y 2 (CtBP1,2), colorectal cancer (CRC), decenoylcarnitine (C16:1), docosahexaenoic (C22:6), diabetic kidney disease (DKD), dichlorodiphenyltrichloroethane (DDT), dehydrogenase (BCKD), endoplasmic reticulum (ER), estimated glomerular filtration rate (eGFR), extracellular vesicles (EVs), fatty acid-binding protein 4 (FABP4), free carnitine (C0), free fatty acids (FFA), gamma-aminobutyric acid (GABA), glycine (GLY), glutaminase 1 (GLS1), growth differentiation factor 8 (GDF8), growth differentiation factor 11 (GDF11), GS??, hexokinase 2 (HK2), high density lipoprotein (HDL), high-resolution mass spectrometry (HRMS), histidine (His), hypoxia Inducible Factor 1α (HIF-1α), isoleucine (ILE), immunoglobulin heavy constant mu chain (IGHM), kininogen (KNG) lactate dehydrogenase A (LDHA), leucine (LEU), leucine + isoleucine (ILE), leucine rich repeat kinase 2 (LRKK2), liquid chromatography (LC), liquid chromatography/mass/mass (LC-MS/MS), low density lipoprotein (LDL), lysophosphatidylcholines (LPC), neurofilament light chain (NF-L), nuclear factor kappa B subunit 1 (NFKB1), nuclear magnetic resonance (NMR), mass spectrometry (MS), member of the class O of forehead (FOXO), MYC proto-oncogene, bHLH transcription factor (c-Myc), octadecanoylcarnitine C18:0, octadecadienyl-L-carnitine (C18:3), octadecenoylcarnitine (C18:1), octanoylcarnitine (C8), operating characteristic curve (AROC), ornithine (ORN), Parkinson’s disease (PD), pharmacogenetics and pharmacogenomics (PGx), phenylalanine (PHE), phenylacetylglutamine (PAGln), phosphocholine lipid species (PC), phosphoethanolamine species (PE), phosphoserines (PS), polyunsaturated fatty acids (PUFA), positron emission tomography (PET), proline (PRO), protein Phosphatase, Mg^2+^/Mn^2+^ Dependent 1K (PPM1K) protein kinase B (PKB), also (Akt), prostate cancer (PC), protein kinase C-delta (PKCδ), PTEN-induced kinase 1 (PINK1), polyunsaturated fatty acids (PUFA),quantitative trait loci (eQTL), rat sarcoma virus (Ras), receptor 2 of human epidermic growth factor (HER2), receptor tyrosine kinase (ROS), target of rapamycin (TOR), serum-free carnitine (C0), short-chain dicarboxylacylcarnitine species (SCDA), single-cell metabolic regulome profiling (scMEP), sirtuin1/6 (SIRT1/6), sphingomyelins (SM), tauopathy transgenic rat model (SHR72), tauopathy mouse model (rTg4510), tetradecenoylcarnitine (C10:2), thiopurine methyltransferase (TPMT), TNF superfamiliy Member 12 (TNFSF12) transcription factors (TF), transcription factor p65 (RELA), transmembrane serine protease 2 (TMPRSS2), triboelectric nanogenerator inductive nanoelectrospray ionization (TENGi MS), ion mobility mass spectrometry (IM-MS), triglycerides (TG), trimethylamine N-oxide (TMAO), tryptophan (trp), tumoral protein 53 (TP53), tumor microenvironment (TME), type 2 diabetes (T2D), type 1 (T1D), tyrosine (TYR), ultra-performance liquid chromatography (UPLC), U.S. Food and Drug Administration (FDA), valine (VAL), very low densityl lipoprotein (VLDL), WAP/kazal/inmunoglobulin/Kunitz and NTR domain-containing protein 2 (WFIKKN2), whole-exon sequencing (WES).
